# Supplementary material for: FLT3-ITD in Children with Early T-cell Precursor (ETP) Acute Lymphoblastic Leukemia: Incidence and Potential Target for Monitoring Minimal Residual Disease (MRD)
Source: Cancers (Basel). 2022 May 17;14(10):2475. doi: 10.3390/cancers14102475 (PMC9139937; doi:10.3390/cancers14102475)
Supplement: Supplementary file 1 [file cancers-14-02475-s001.zip › cancers-1644343-supplementary.pdf]

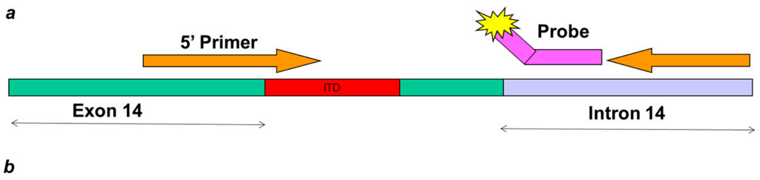


**Figure S1.** Model of probe and primers design for detection of Minimal Residual Disease (MRD) in children with Early T-precursor Acute Lymphoblastic Leukemia. In (**a**), it is showed how we designed two different common reverse primers and one fluorescent probe, in order to cover any duplication. Position of Forward (5′) primer (FP) and Reverse (3′) primer (RP) is indicated with orange arrows, positioned in opposite direction. The FP [5′- TGTTTCTGCAGCATTTCTTTTCCATTG-3′ (27 bp)], was designed across exon 14 (**b**) and the ITD region in order to increase the specificity. The RP [5′- TGTTTCTGCAGCATTTCTTTTCCA-3′ (24 bp)], was designed based on the sequences of Intron 14 (**b**). The probe [5′- AGAGTTTGGTAAGAATGGAATGT-3′ (23 bp)] MGB on intron 11 were used for duplication occurring in exon 11.

**Table S1.** Characteristics of children with ETP-ALL enrolled in three consecutive AIEOP-ALL protocols and screened for detection of FLT3-ITD.

| | ***FLT3* pos** | | ***FLT3* neg** | | **TOTAL** | |
|---|---|---|---|---|---|---|
| | *N* | % | *N* | % | *N* | % |
| Total *n.* of patients | 10 | *13.0* | 67 | *87.0* | 77 | |
| GENDER | | | | | | |
| Male | 9 | 90.0 | 45 | 67.2 | 54 | 70.1 |
| Female | 1 | 10.0 | 22 | 32.8 | 23 | 29.9 |
| AGE | | | | | | |
| 1–5 yrs | 2 | 20.0 | 12 | 17.9 | 14 | 18.2 |
| 6–9 yrs | 3 | 30.0 | 18 | 26.9 | 21 | 27.3 |
| 10–17 yrs | 5 | 50.0 | 37 | 55.2 | 42 | 54.5 |
| WBC | | | | | | |
| <20000 | 1 | 10.0 | 40 | 59.7 | 41 | 53.2 |
| 20–100000 | 7 | 70.0 | 16 | 23.9 | 23 | 29.9 |
| ≥100000 | 2 | 20.0 | 11 | 16.4 | 13 | 16.9 |
| Pred RESPONSE | | | | | | |
| Good | 2 | 20.0 | 37 | 55.2 | 39 | 50.6 |
| Poor | 8 | 80.0 | 30 | 44.8 | 38 | 49.4 |
| Response to Ia | | | | | | |
| CR | 9 | 90.0 | 53 | 81.5 | 62 | 82.7 |
| Resistant | 1 | 10.0 | 12 | 18.5 | 13 | 17.3 |
| Not known/not applicable | | | 2 | | 2 | |
| MRD stratification | | | | | | |
| Standard | 0 | | 2 | 3.0 | 2 | 2.6 |
| Medium | 2 | 20.0 | 24 | 35.8 | 26 | 33.8 |
| High | 2 | 20.0 | 7 | 10.4 | 9 | 11.7 |
| Not known | 6 | 60.0 | 34 | 50.7 | 40 | 51.9 |
| FINAL RISK | | | | | | |
| Standard | 0 | | 5 | 7.5 | 5 | 6.5 |
| Medium | 0 | | 20 | 29.8 | 20 | 26.0 |
| High | 10 | 100 | 42 | 62.7 | 52 | 67.5 |
| Protocol | | | | | | |
| ALL 2000/R2006 | 4 | 40.0 | 29 | 43.3 | 33 | 42.9 |
| ALL 2009 | 6 | 60.0 | 38 | 56.7 | 44 | 57.1 |
| Flow day +15 (only ALL 2009) | | | | | | |
| <0.1% | 0 | | 4 | 11.1 | 4 | 9.5 |
| ≥0.1%–<10% | 2 | 33.3 | 9 | 25.0 | 11 | 26.2 |
| ≥10% | 4 | 66.7 | 23 | 63.9 | 27 | 64.3 |
| Not known | 0 | | 2 | | 2 | |

Yrs: years; WBC: white blood cell; Pred: prednisone; Ia: Induction Ia; CR: complete remission; MRD: minimal residual disease.

**Table S2.** Features of FLT3-ITD RQ-PCR in children with ETP-ALL.

| Pt | ITD lenght | random nt | slope | coeff corr | QR | SR |
|---|---|---|---|---|---|---|
| 1 | 74 | 10 | 3.29 | 0.99 | $1 \times 10^{-3}$ | $1 \times 10^{-4}$ |
| 2 | 43 | 5 | 4.23 | 1.00 | $1 \times 10^{-4}$ | $1 \times 10^{-4}$ |
| 3 | 48 | 4 | 3.54 | 0.99 | $5 \times 10^{-4}$ | $1 \times 10^{-5}$ |
| 4 | 32 | 4 | 4.22 | 0.98 | $5 \times 10^{-4}$ | $1 \times 10^{-5}$ |
| 5 | 71 | 4 | 4.35 | 0.98 | $5 \times 10^{-4}$ | $1 \times 10^{-5}$ |
| 6 | 26 | 0 | 3.81 | 0.99 | $1 \times 10^{-4}$ | $1 \times 10^{-5}$ |
| 7 | 28 | 2 | 3.64 | 1.00 | $5 \times 10^{-4}$ | $1 \times 10^{-5}$ |
| 8 | 65 | 1 | 3.89 | 1.00 | $1 \times 10^{-4}$ | $1 \times 10^{-5}$ |
| 9 | 63 | 9 | 2.92 | 1.00 | $1 \times 10^{-5}$ | $1 \times 10^{-5}$ |
| 10 | 254 | 21 | 4.26 | 1.00 | $1 \times 10^{-5}$ | $1 \times 10^{-5}$ |
| Mean | 70.4 | 6 | 3.82 | 0.99 | $3.3 \times 10^{-4}$ | $2.8 \times 10^{-5}$ |
| Median | 55.5 | 4 | 3.81 | 0.99 | $5 \times 10^{-4}$ | $1 \times 10^{-5}$ |

Features of ITD length, random nucleotides in each patient and RQ-PCR approach for detection of FLT3-ITD. The quantitative range (QR) was between $5 \times 10^{-4}$–$1 \times 10^{-5}$ and sensitivity range was between $1 \times 10^{-4}$–$1 \times 10^{-5}$. Pt patient; nt nucleotide; Coeff Corr: Correction Coefficient; QR quantitative range; SR sensitive range;

**Table S3.** Features of FLT3-ITD RQ-PCR in non-ETP-ALL.

| Pt | ITD lenght | random nt | slope | coeff corr | QR | SR |
|---|---|---|---|---|---|---|
| 1 | 30 | 17 | 4.03 | 0.99 | $5 \times 10^{-4}$ | $1 \times 10^{-4}$ |
| 2 | 24 | 2 | 3.98 | 0.99 | $1 \times 10^{-4}$ | $1 \times 10^{-4}$ |
| 3 | 63 | 3 | 4.25 | 0.99 | $1 \times 10^{-4}$ | $1 \times 10^{-5}$ |
| 4 | 36 | 5 | 3.89 | 1 | $5 \times 10^{-4}$ | $1 \times 10^{-5}$ |
| Mean | 38.25 | 6.75 | 4.04 | 0.99 | $3 \times 10^{-4}$ | $5.5 \times 10^{-5}$ |
| Median | 33 | 4 | 4 | 0.99 | $3 \times 10^{-4}$ | $5 \times 10^{-5}$ |

FLT3-ITD MRD monitoring was also performed in 4 cases with FLT3-ITD positive no-ETP ALL. Mean length of the ITD was 38.25 nucleotides (nts) (range 24–63), with a mean of 6.75 randomly inserted nts (range 3–17). Standard curves performed by 10-fold dilutions in DNA from PB Healthy Donor, showed a quantitative range of at least $5 \times 10^{-4}$ in two cases and $1 \times 10^{-4}$ in the other two cases. Sensitivity of the assay was $1 \times 10^{-5}$ in two and $1 \times 10^{-4}$ in the other two cases, respectively. Pt patient; nt nucleotide; Coeff Corr: Correction Coefficient; QR quantitative range; SR sensitive range.

**Table S4.** Features of RQ-PCR using T-cell Receptor rearrangements in ETP-ALL.

| Cases | Rearrangement | QR | SR | Ct undiluted | Ct background | Slope | T° | C. Coeff. |
|---|---|---|---|---|---|---|---|---|
| ETP-Case 4 | TR Vδ1Jδ1 | $1 \times 10^{-4}$ | $1 \times 10^{-4}$ | 22.88 | 40.91 | −3.82 | 61.0 | 1.00 |
| | TR Vγ9Jγ2.3 | $1 \times 10^{-3}$ | $5 \times 10^{-4}$ | 24.12 | 36.91 | −3.47 | 61.0 | 0.99 |
| ETP-Case 9 | TR Vβ2.1Jβ2.2 | $1 \times 10^{-4}$ | $1 \times 10^{-4}$ | n.a. | n.a. | n.a. | 61.0 | n.a. |
| | TR Vδ1Jδ1 | $1 \times 10^{-4}$ | $1 \times 10^{-4}$ | n.a. | n.a. | n.a. | 61.0 | n.a. |
| ETP-Case 3 | TR Vγ9Jγ2.3 | $5 \times 10^{-4}$ | $1 \times 10^{-5}$ | 23.47 | 50.00 | −3.63 | 60.5 | 0.99 |
| | TR Vδ1Jδ1 | $1 \times 10^{-3}$ | $1 \times 10^{-4}$ | 22.95 | 50.00 | −3.75 | 60.5 | 0.99 |

TR T-cell Receptor; QR quantitative range; SR sensitive range; Ct cycle threshold; T° temperature in Celsius; C. coeff correction coefficient; n.a. not available.
